# COVID-19, Villitis and Placenta in Pregnancy

**DOI:** 10.5146/tjpath.2020.01520

**Published:** 2022-05-19

**Authors:** Viroj Wiwanitkit

**Affiliations:** Dr. DY Patil University, Department of Community Medicine, Pune, Maharashtra, India

**Dear Editor,**

We would like to correspond and share ideas on the publication “Movement Disorders in COVID-19: Whither Art Thou? (1).” Ozer et al. noted that “We hypothesize that VUE may represent a maternal anti-viral immune response, in this case to SARS-CoV-2 (1).” Theoretically, the virus infection in pregnancy might affect the placenta. Regarding COVID-19, it is proven that the placental barrier can prevent transplacental migration of the virus based on its nanostructure (2). This means the circulating virus during viremia in a pregnant woman might rod into the placenta without crossing the placenta. It is no doubt that there might be an immune mechanism at the placenta that can lead to villitis. If there is no underlying placenta pathology, there should be no transplacental transmission to the fetus in utero (2,3).

## Conflict of Interest

The author declares no conflict of interest.
